# A UAV-Based Eddy Covariance System for Measurement of Mass and Energy Exchange of the Ecosystem: Preliminary Results

**DOI:** 10.3390/s21020403

**Published:** 2021-01-08

**Authors:** Yibo Sun, Junyong Ma, Bilige Sude, Xingwen Lin, Haolu Shang, Bing Geng, Zhaoyan Diao, Jiaqiang Du, Zhanjun Quan

**Affiliations:** 1 Institute of Ecological Environment, Chinese Research Academy of Environmental Sciences, Beijing 100012, China; sunyb68@163.com (Y.S.); mjy172404707@me.com (J.M.); diaozhy@126.com (Z.D.); dujq@craes.org.cn (J.D.); 2 State Key Laboratory of Environmental Criteria and Risk Assessment, Beijing 100012, China; 3 State Environmental Protection Key Laboratory of Regional Ecological Processes and Functions Assessment, Beijing 100012, China; 4 Integrated Ecological Observation and Research Station of Jinggangshan, Jinggangshan 343699, China; 5 Collage of Geography and Environment Science, Zhejiang Normal University, Jinhua 321004, China; linxw@zjnu.edu.cn; 6 Key Laboratory of Digital Earth Science, Aerospace Information Research Institute, Chinese Academy of Sciences, Beijing 100028, China; hl.shang@hotmail.com; 7 Research Institute for Eco-Civilization, Chinese Academy of Social Sciences, Beijing 100028, China; gengbing46@163.com; 8 Chinese Research Academy of Environmental Sciences, Beijing 100012, China

**Keywords:** unmanned aerial vehicle, airborne eddy covariance, wind speed measurement, turbulent fluxes

## Abstract

Airborne eddy covariance (EC) measurement is one of the most effective methods to directly measure the surface mass and energy fluxes at the regional scale. It offers the possibility to bridge the scale gap between local- and global-scale measurements by ground-based sites and remote-sensing instrumentations, and to validate the surface fluxes estimated by satellite products or process-based models. In this study, we developed an unmanned aerial vehicle (UAV)-based EC system that can be operated to measure the turbulent fluxes in carbon dioxides, momentum, latent and sensible heat, as well as net radiation and photosynthetically active radiation. Flight tests of the developed UAV-based EC system over land were conducted in October 2020 in Inner Mongolia, China. The in-flight calibration was firstly conducted to correct the mounting error. Then, three flight comparison tests were performed, and we compared the measurement with those from a ground tower. The results, along with power spectral comparison and consideration of the differing measurement strategies indicate that the system can resolve the turbulent fluxes in the encountered measurement condition. Lastly, the challenges of the UAV-based EC method were discussed, and potential improvements with further development were explored. The results of this paper reveal the considerable potential of the UAV-based EC method for land surface process studies.

## 1. Introduction

The land surface and the climate system interact through a series of bidirectional feedback, including the surface fluxes in mass and energy, momentum, boundary layer dynamics, and boundary layer properties [1,2]. Substantial worldwide efforts have been undertaken to identify the spatial and temporal variabilities in the surface fluxes, resulting in remarkable achievements. However, an observational spatial scale mismatch exists in our understanding of surface flux exchange between the local and global scales [3,4]. Flux observation based on the ground-based eddy covariance (EC) or Optical-Microwave Scintillometer techniques can provide reliable and temporally continuous observations of surface–atmosphere fluxes’ exchange, but the obvious limitations are the poor spatial representation and low distribution density [5,6,7]. Nowadays, the regional and global surface fluxes can be estimated using remote sensing or process-based models, but these methods must be validated or evaluated using true observations [8,9]. However, the direct observations of surface fluxes at similar scales to remote-sensing or process-based models are lacking, which restricts the study and development of simulations of regional and global surface fluxes [10]. Therefore, the regional-scale observation is considered the missing scale in the study of global surface flux [3,11,12]. 

In this context, airborne flux measurements based on the EC method can be used to directly measure the surface fluxes on the regional scale, showing potential to close the existing observational scale gap [13]. The airborne EC method is an established flux measurement technique that has been used extensively in recent decades and has been validated against tower-based EC and large aperture scintillometer flux measurement methods [13,14,15,16]. The major advantage of airborne EC is the ability to measure turbulent fluxes that are more spatially representative than ground-based EC measurements and that have higher temporal resolution and accuracy than those estimated from models [17]. At present, small environmental research aircraft (SERA), such as the Sky Arrow 650 environmental research aircraft (ERA) or Twin Otter, represent the most advanced airborne flux technology and have been used in numerous large field experiments [18,19]. These airborne EC measurements offer the opportunity to investigate surface fluxes on the regional scale and complement ground-based observations by providing insight into the spatial variability information of surface fluxes [20]. However, flux measurements based on manned aircraft have several shortcomings. The operation and maintenance of manned research aircrafts are expensive [21], and low-altitude flights in the near surface layer are strictly limited due to pilot safety considerations [22,23]. Regulatory issues, legal issues, and cost issues restrict the widespread application of airborne flux measurements.

Recently, the advances in unmanned aerial vehicle (UAV) technology, as well as micro-electro-mechanical systems (MEMS), global positioning system (GPS), and batteries, have enabled the UAV to carry smaller, faster and more energy-efficient observation instruments for ecosystem studies [12,24]. Compared to manned aircraft, unmanned and automatically operating aircrafts have minimal logistical requirements (e.g., no airport is necessary), and are more flexible, cheaper to operate and maintain, and are less disturbance to turbulence. They can fly within the lower part of the atmosphere boundary layer and in regions that are difficult or dangerous for manned aircraft [25]. These advantages motivated the development of UAV-based turbulent fluxes measurement techniques to overcome the drawbacks of manned aircraft.

Attempts to measure the turbulent fluxes using UAVs were proposed as early as the 1990s [26]. At that time, the mobile flux platform (MFP) was relatively large and heavy, and therefore required large UAVs upon which to install the observation sensors. These large UAVs were retrofitted from military aircraft (e.g., Global Hawk or Ikana) or ultralight manned aircraft (e.g., Challenger II from Canada) for scientific missions and have the same limitations as manned aircrafts [27]. During the last decade, with the developments in autopilot and the ongoing miniaturization of relevant sensors, UAVs are increasingly used to measure turbulent fluxes. Several types of UAV-based turbulent flux measurement systems have been developed and deployed. According to the take-off weight and payload capacity of the aircraft, these UAVs can be classified as small, medium, or large, with each having different sensor suites [28]. The take-off weight of small UAVs is less than 1 kg; they have smaller payload capacity and endurance compared with the other two categories. The typical small-type UAV-based flux measurement system is the small unmanned meteorological observer (SUMO), with an overall take-off weight of around 0.6 kg and endurance of around 0.5 h. The SUMO was initially designed for measuring the atmosphere profiles of temperature, humidity, and wind (based on the ‘no-flow sensor’ algorithm) [29], and then improved for the measurement of a 3D turbulent flow and sensible heat flux by equipping an inertial measurement unit (IMU), a fast temperature sensor, and a 5-hole probe [30]. The SUMO has been used in multiple campaigns, and helped to increase our understanding of dynamic atmospheric processes by complementing ground-based observation and numerical models [24,31,32]. However, limited by the small payload capacity and endurance, small UAVs can only obtain a few observation parameters for turbulence measurement, and cannot resolve large-scale eddies, which significantly contribute to turbulence energy. Moreover, during flight, small UAVs are susceptible to interaction with the turbulence, which leads to the need for an additional method (e.g., filtering algorithms) to restrain the measurement error [24,33]. 

The medium UAV-based flux measurement systems include aircrafts with a take-off weight between 1 and 10 kg and endurance of around 1 h, such as the Tempest, meteorological mini unmanned aerial vehicle (M^2^AV), multi-purpose automatic sensor carrier (MASC), Skywalker X6, BLUECAT5, and Objet Volant Leger Instrumenté–Turbulence Atmosphérique (OVLI-TA) [34,35,36,37,38]. The endurance and payload capacity of these medium UAVs make it possible to carry more and better sensors for turbulence measurements than small UAVs. For instance, the M^2^AV (6 kg takeoff weight) is equipped with a miniaturized 5-hole probe, two temperature sensors (a fast temperature sensor thermocouple, and an accurate but slow temperature sensor (Vaisala HMP 50)), and a humidity sensor (Vaisala HMP 50) for measurement of atmosphere profile as well as sensible heat flux [39]. The performance of M^2^AV has been well documented by comparison with various methods including meteorological tower and remote-sensing observations, and has been deployed in many field campaigns [39,40,41]. For the medium UAVs, their cost is moderate and they are still easy to operate. However, the observation ability is still limited: only a few turbulent flux parameters (e.g., momentum and sensible heat flux) can be measured due to the lack of a sufficient payload capacity. 

Large UAVs provide significantly higher endurance and payload capabilities than the two other types. They include vehicles with a take-off weight of more than 10 kg and an endurance of more than 2 h, and they have a similar observation ability to lightweight manned aircrafts. At present, the large UAVs that are capable of turbulent flux measurements include UMARS2, Manta, ScanEagle, and Application of Lightweight Aircraft for Detecting IN situ Aerosol (ALADINA) [12,42]. The Manta (27.7 kg takeoff weight) and ScanEagle (22 kg takeoff weight) were developed for direct flux measurements of momentum and of sensible and latent heat fluxes within the terrestrial and marine atmospheric boundary layers [43,44]. These platforms are both equipped with two different types of payloads with different measurement objectives, including a flux payload and radiometric payload. The flux payloads consist of a 9-hole turbulence probe for measurement of relative wind speed, a krypton hygrometer for fast-response water vapor, an optic temperature sensor for fast-response temperature, a slower-response temperature and relative humidity probe for compensation for the fast-response sensors (temperature and moisture), a nadir-looking LiDAR altimeter for maintaining a constant relative height during flight, and a highly accurate Global Positioning System/Inertial Navigation System (GPS/INS) integrated navigation system for measurement of aircraft attitude and velocity [43]. An onboard computer controls data acquisition, data logging, and synchronization of all the sensors, as well as the communication with the ground control station. The operation of large UAVs is more complex than the other two types of UAV, for example, requiring a runway for takeoff and landing, and highly specialized ground support staff for the mission. Nevertheless, large UAVs provide a suitable platform for turbulent flux measurements due to their high payload capability and long flight, as well as the reduced operational costs, and are less disturbance to turbulence than manned aircraft.

The key to the successful achievement of airborne flux measurements requires a fast response instrumentation, capable of measuring concentrations with a time resolution that is sufficient to resolve the turbulent fluctuations affecting the flux transport, and with an accuracy that can measure the differences in the concentration of the parameter depending on the direction in which the air is moving [14]. Nowadays, many research-grade measurements devices that once required manned aircraft are now available for unmanned aerial vehicles. For example, the wind speed, as the most important variable for EC measurements, requires accurate measurement of the 3D wind speed with respect to the wind probe, as well as the velocity and attitude of the aircraft with respect to the earth [43]. This was achieved by a multi-hole probe paired with a GPS/INS integrated navigation system. SUMO, M^2^AV, MASC, ALADINA, Skywalker X6, and BLUECAT5 are all equipped with a custom or commercial lightweight five-hole probe and GPS/INS to measure the wind vector, and achieved promising results. The Manta and ScanEagle systems developed by Reineman et al. (2013), equipped with a custom nine-hole probe and combined with a high-precision GPS/INS, provided highly precise wind measurements with a similar performance to that reported with the Best Atmospheric Turbulence (BAT) probe on a manned aircraft [37]. In addition, the temperature sensors could be easy installed on UAVs, and temperature could be quickly and accurately measured by coupling a fast temperature sensor (thin thermocouple or platinum resistance thermometer) and an accurate but slow response temperature sensor (thermistor) [45]. Therefore, for the UAV-based EC measurements, a multi-hole probe and temperature probe could be equipped for measurement of momentum and sensible heat fluxes, especially for the small and medium UAVs with limited payloads. For the measurements of latent heat and CO_2_ fluxes, it is difficult to deploy fast gas analyzers on small and medium UAVs due to the size and mass of gas analyzers being relatively large. Only large UAVs can carry fast gas analyzers to determine the latent heat or/and CO_2_ fluxes, such as the Manta and ScanEagle, which carry a repackaged KH2O krypton hygrometer for the determination of latent heat flux. For the radiation fluxes (net flux, photosynthetically active radiation flux, etc.), which are the main force driving the ecosystem process, UAVs can seldom measure turbulent and radiation fluxes simultaneously due to the limitations in the payloads. This is the main drawback of UAV-based flux measurement methods compared to the current advanced manned airborne flux technology. The current method involves two different types of payload to measure the turbulent fluxes or radiation fluxes, such as Manta and ScanEagle [43]. In summary, unmanned aircraft platforms offer distinct advantages over manned aircraft in their ability to safely perform measurements in situations which are dangerous for manned aircraft and with significantly reduced operational and maintenance costs, providing an ideal platform for airborne flux measurements. However, the mismatch between the payload capacity of UAVs and the flux instrumentation results in the current UAVs only being able to obtain several turbulence variables; a large gap remains in the flux measurement ability between UAVs and manned aircrafts. Fortunately, the introduction of high payload vertical take-off and landing (VTOL) fixed-wing platforms and more easily carried CO_2_/H_2_O gas analyzers has enabled UAVs to carry more comprehensive sensors, similar to manned research aircrafts, to measure the fluxes. Therefore, a highly integrated UAV-based flux measurement system must be developed for the simultaneous observation of turbulent fluxes and radiation fluxes, which was the central purpose of this study.

In this paper, we describe a high payload vertical takeoff and landing fixed-wing UAV for measuring turbulent fluxes including sensible heat, latent heat, and CO_2_, as well as radiation fluxes including net radiation and upward- and downward-looking photosynthetically active radiation (PAR). The performance of turbulent fluxes measurements of this UAV-based EC system was evaluated by conducting a wind tunnel test, a ground-based comparison test, and three in-flight comparison tests. This paper presents the preliminary results of these tests for investigating the measurement reliability of wind speed and turbulent fluxes in this system.

## 2. System Description 

### 2.1. UAV

**Figure 1:**
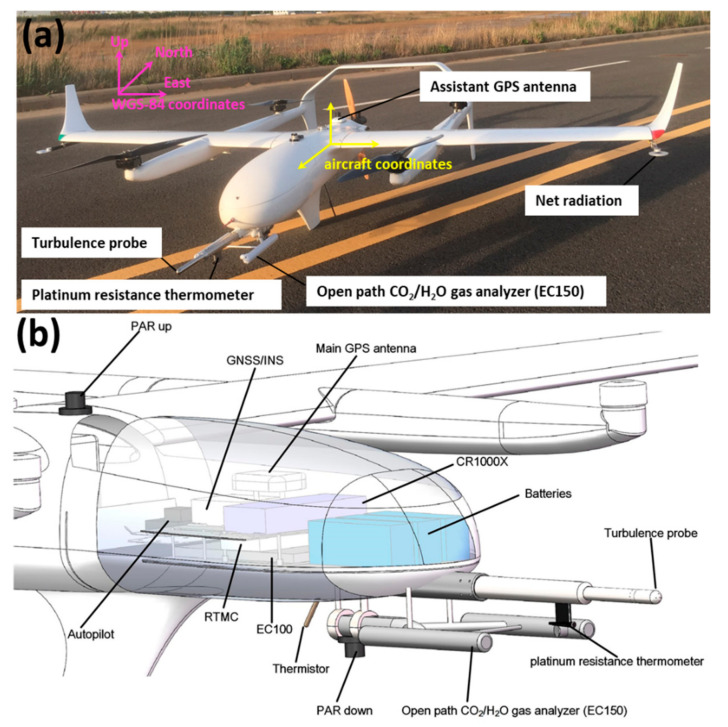
The developed unmanned aerial vehicle (UAV)-based flux measurement system with the coordinate system information (**a**) and the computer graphic of the carried scientific payloads (**b**).

The unmanned aerial platform we used is a fuel-powered VTOL fixed-wing UAV (F-EYE UAV Technology, Tianjin, China), offering a high payload capacity, and a durable and aerodynamic platform with extended flight endurance (Figure 1). This airframe was selected as the platform of the UAV-based flux measurement system as it has minimal requirements for the takeoff locations, a high payload capacity, and easy modifications. The detailed specifications and capabilities of this aircraft are provided in Table 1. It has a wingspan of 3.7 m, a fuselage length of 2.85 m, and a maximum take-off weight of 60 kg, of which up to 10 kg is designated for scientific payloads. The VTOL fixed-wing UAV has the advantages of both the fixed wind and the rotor wing aircraft, which can take off in vertical mode, hover in place, transition into horizontal flight, transition back to vertical flight, and land in vertical mode. This type of aircraft does not need runways or launch-recovery equipment, and is suitable for flight missions in complicated environments.

Control of this UAV is completely autonomous, including takeoff and landing. Pilots have the option to enable manual or semi-manual control in an emergency. During the flight, the UAV system is controlled by an on-board autopilot, typically with a cruising groundspeed of around 28 to 31 m/s, and with flight ceilings of up to 3.8 km. This autopilot allows precise control of the position of the aircraft during flights with a horizontal accuracy of 1 cm and vertical accuracy of 3 cm (provided by the manufacturer). The engine is mounted in a pusher configuration, allowing the multi-hole probe to be directly installed on the aircraft’s nose, which could minimize the airflow contamination due to the interference effects from the airframe. The battery system for the payloads consists of two batteries with a combined capacity of 10 Ah, which enable the measurement system to function for up to 4 h.

### 2.2. Scientific Payloads

**Figure 2:**
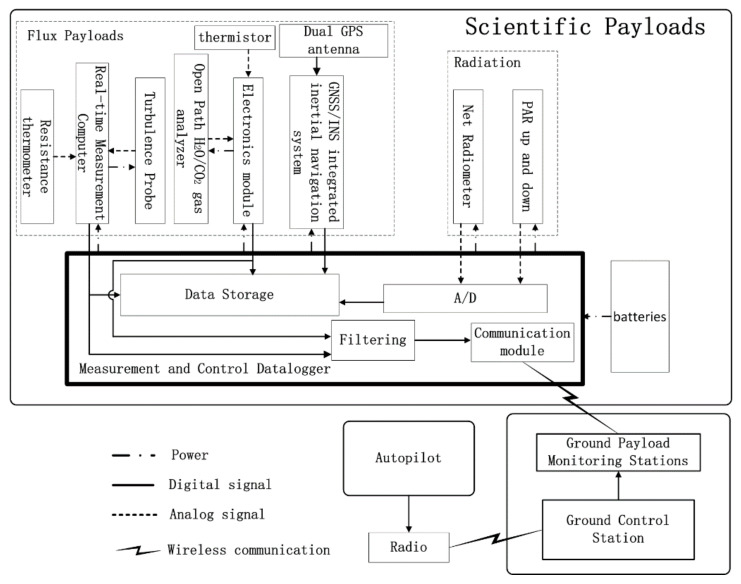
Diagram of the developed UAV-based EC system. Solid arrows represent the digital signal, dashed arrows represent the analog data (measured with the analog-to-digital boards), dotted arrows represent the power supply, and the lightning mark represents radio communication.

Due to the high payload capacity (10 kg) and the aerodynamic configuration of the selected UAV platform, the instruments, including those for flux and radiometric measurements, could be integrated together for scientific measurement. Table 2 provides detailed sensor information with accuracies and sampling frequency. The scientific payloads include flux and radiation payloads, which are designed to be isolated from the flight control system to ensure that any problems from electrical or software in the scientific instrumentation would not jeopardize the safety of the aircraft. The system components and connectivity of the developed UAV-based EC system are presented in Figure 2. 

The flux payloads include a 5-hole turbulence probe, an open-path CO_2_/H_2_O infra-red gas analyzer (IRGA), a dual-antenna integrated inertial navigation system (GPS/INS), and two temperature sensors. The 5-hole turbulence probe, as the critical sensor for turbulent flux measurement, was made by the Simtec AG (Basel, Switzerland) and consists of a five-hole probe and a real-time measurement computer (RTMC). The 5-hole probe was used to quickly measure the wind velocities in three dimensions with respect to the UAV through measurements of the relative true airspeed, the attack (α) and sideslip (β) angles. The RTMC was used as the data-parsing and device-maintenance interface for the 5-hole probe. The 5-hole probe was rigidly connected beneath the shockwave point of the nose of the UAV with a flush-mounted aluminum flange. It has a diameter of 22 mm, length of 433.5 mm and weight of less than 1 kg. The 5-hole probe measures static and dynamic pressures through small holes at its side and tip, and then converts these measurements to relative wind speed. At the tip, a 22 mm stainless steel hemisphere has a center port for total pressure measurement, and four additional ports arranged in a cruciform pattern for attack (α) and sideslip (β) angle measurements. At the side, 24 ports sharing a common manifold for static pressure measurement are located 47.5 mm back from the tip. A fast temperature sensor platinum resistance thermometer (PT 100 with tolerance class Y, Simtec AG, Basel, Switzerland), which is covered by a shield, is installed underneath the probe tube for measurement of the fast temperature fluctuations. The attitude, location and groundspeed of the UAV are measured by an integrated inertial navigation system (GPS/INS), which outputs Kaman-filtered data, including the attitude angles (roll ϕ, yaw ψ, and pitch θ), GPS position, altitude, and ground speeds of the UAV in the geographic coordination system (WGS-84 coordinate system in this paper, Figure 1b). As the heading information is one of the main sources of errors in wind calculation [28,46], we adopted a dual-antennae receiver with an integrated inertial navigation system (BD992-INS, Trimble, Sunnyvale, CA, USA), which has a heading accuracy of 0.1° (with 1 m baseline) and roll/pitch accuracy of 0.1°. The GPS/INS device is installed at the center of gravity (CG) of the UAV, and two GPS antennae are flat-mounted along the longitudinal axis of the UAV with a baseline of 1.1 m. The absolute densities of water vapor and carbon dioxide are measured by a CO_2_/H_2_O open-path gas analyzer (EC150, Campbell, Logan, UT, USA), which is fixed adjacent to the turbulence probe below the nose of the UAV. Rubber vibration damping was used at the contact point between the EC150 and the bottom surface of the fuselage for reducing the resonance effect from the engine and propeller. The gas analyzer component also includes an electronic module (EC100, Campbell, Logan, UT, USA), which is used to collect and output the measurement data from the EC150. Additionally, a slow-response but high-absolute-accuracy temperature probe (thermistor) is connected to the EC100 and extends downward from the bottom surface of the UAV. The two temperature sensors are coupled using a complementary filtering for obtaining long-term stability and high-accuracy temperature data. The sampling frequency of all the flux payloads is 50 Hz; accordingly, a horizontal spacing of 0.6 m between 50 Hz measurements in no-wind conditions can be achieved when the UAV flies at a 30 m/s ground speed. In this way, eddies of wavelength larger than 1.2 m can be resolved (Nyquist’s theorem). 

The radiometric payloads include a net radiometer (NR-LITE2, Campbell, Logan, UT, USA) and two PAR radiometers (PQS 1, Zipp and Zonen, Delft, Netherlands) that look upward and downward. The net radiometer is mounted on the left aerofoil tip for measurement of the algebraic sum of incoming and outgoing all-wave radiation (both short- and long-wave components). A fake net radiometer is mounted on the symmetrical position of the right aerofoil tip to maintain the weight balance and the aerodynamic symmetry of the UAV. Two PAR radiometers, one each on the upper and lower surface of the UAV, are mounted for measurement of the upwelling and the downward PAR. The measurement frequency of the radiometric payloads is 10 Hz.

The inner structure of the developed UAV-based flux measurements system is shown in Figure 1b. A datalogger (CR1000X, Campbell, Logan, UT, USA) is used as the data acquisition computer for synchronization and logging of digital and analog data from the scientific payloads. It receives a power supply from the batteries on-board and serves as the power distribution to the scientific payloads. The datalogger is also equipped with a communication port, the 1-s averaged static pressure and CO_2_/H_2_O concentration data from the RTMC and EC100 are transmitted to the ground monitoring station for keeping the ground operators informed of atmospheric conditions and the main sensor status. 

## 3. Methods

### 3.1. Calibration and Validation of the 5-Hole Turbulence Probe

The wind tunnel calibration of the five-hole turbulence probe was performed by the probe manufacturer, and a precision interpolation table was generated for deriving calibrated α, β, and dynamic (q) and static ($p_{s}$) pressure. These parameters are required to characterize the local wind flow relative to the UAV. During the wind tunnel calibration, the amplitudes of α and β varied up to ±20° to simulate the largest envelope of expected flight conditions, and the calibrated airspeed was set to 20–40 m/s (the mission speeds are typically around 30 m/s). During flight observation, the interference effects between the airframe and 5-hole turbulence probe were neglected due to the tubing length of 433.5 mm between the turbulence probe tip and the nose of the UAV, which was long enough to avoid the influence of the turbulence deflection around the airframe. 

The manufacturer’s calibrations were performed with the 5-hole probe alone. For validation of the synthesized wind measurement performance of the developed UAV-based flux measurement system, we performed an additional validation procedure by mounting the 5-hole probe on the UAV fuselage (minus wings), and compared the results with the conventional ground-based sonic anemometer on the ground (Section 4.1).

### 3.2. Geo-Referenced Wind Calculation

The EC technique requires the time series of both the transported scalar quantity and the transporting turbulent wind, each measured at a sufficient frequency to resolve the eddies [47]. For the airborne EC method, the turbulent wind is measured by combining the measurement from the turbulence probe (i.e., 5-hole turbulence probe in this study) and the GPS/INS. The geo-referenced wind (U) is the vector sum of the wind speed from the probe (${\hat{U}}_{a}$) with respect to the UAV and the UAV’s motion ($U_{p}$) with respect to the earth, which is defined as [47]

(1) $$U\left(t\right)=G\left(t\right)\left[{\hat{U}}_{a}\left(t\right)+{\hat{w}}_{p}\left(t\right)\times \hat{r}\;\right]+\;U_{p}\left(t\right),$$

where the unadorned symbols in Equation (1) are in the geographic coordinate system (x to east, y to north, and z opposite to gravity), ^ denotes the aircraft coordinates, which is a right-hand orthogonal system with $\hat{x}$ directed forward, $\hat{y}$ directed toward the port wing, $\hat{z}$ directed upward, and the center is the CG of the UAV (Figure 1b). G is the transformation matrix, ${\hat{w}}_{p}$ is the rotational velocity of the aircraft in aircraft coordinates, and $\hat{r}$ is the position vector from the CG of the UAV to the sensing tip of the turbulence probe in aircraft coordinates. The cross-product term (${\hat{w}}_{p}\left(t\right)\times \hat{r}$) in Equation (1) describes the transformation due to the spatial separation between the turbulence probe and the CG of UAV, which could be negligible since $\hat{r}$ is only several centimeters in our UAV system [48]. 

For the developed UAV-based EC system, the relative wind speed (${\hat{U}}_{a}$) is measured by the 5-hole turbulence probe using an array of pressure ports on its hemispherical tip and internal differential pressure sensors. The 5-hole probe outputs the calibrated static pressure ($p_{s}$), dynamic pressure (q), total air temperature ($T_{obs}$), and the attack (α) and sideslip (β) angles after being resolved by the RTMC. Firstly, for calculating the relative wind velocity (${\hat{U}}_{a}$), the Mach number (M) is calculated from the dynamic and static pressure as

(2) $$M^{2}=\frac{2}{\gamma -1}\left[{\left(1+\frac{q}{p_{s}}\right)}^{\left(\gamma -1\right)/\gamma }-1\right]$$

where $\gamma =c_{pm}/c_{vm}$ is the ratio of the specific heats at constant pressure and constant volume (1.4 for air). During the flight, the measured air temperature ($T_{obs}$) is contaminated by the adiabatic heating of air decelerating at the temperature probe. The ambient temperature (T) can be calculated from the measured air temperature ($T_{obs}$) using the follow equation [47]

(3) $$T=\;T_{obs}{\left(1+\frac{M^{2}R_{m}}{2c_{vm}}\varepsilon_{r}\right)}^{-1},$$

where $R_{m}$ is the gas constant for moist air, $c_{vm}$ is the associated specific heat at constant volume and $\varepsilon_{r}$ is the non-dimensional temperature recovery factor, with w provided by the manufacturer ($\varepsilon_{r}=\;0.98$ in this paper). Then, the true airspeed ($\left|{\hat{U}}_{a}\right|$) can be calculated from the ambient temperature (T), Mach number (M) and the gas constant for moist air ($R_{m}$), which can be expressed as

(4) $$\left|{\hat{U}}_{a}\right|=M\sqrt{\gamma R_{m}T},$$

Lastly, incorporating the measured attack and sideslip angles, the relative wind components with respect to the aircraft can be calculated as

(5) $${\hat{U}}_{a}=\left[\begin{array}{c} {\hat{u}}_{a}\\ {\hat{v}}_{a}\\ {\hat{w}}_{a} \end{array}\right]=\frac{\left|{\hat{U}}_{a}\right|}{D}\;\left[\begin{array}{c} -1\\ \tan\beta \\ \tan\alpha \end{array}\right],$$

with a normalization factor

(6) $$D=\sqrt{1+\tan^{2}\alpha +\tan^{2}\beta },$$

In Equation (1), the rotation matrix G from the airplane to earth coordinates is generated from sequential roll (ϕ), pitch (θ) and heading (ψ), which are measured by the GPS/INS. Offset corrections ($\varepsilon_{\phi }$, $\varepsilon_{\theta }$, and $\varepsilon_{\psi }$) are introduced here to correct for the possible misalignment between the turbulence probe and the CG of the UAV. These correction constants were determined via dedicated flight maneuvers in the Section 4.2. The velocity of the UAV ($U_{p}$) is measured by the GPS/INS directly. The detailed process to calculate the wind speed from the aircraft platform can be found in the literatures (e.g., Lenschow 1986; Ven den Kroonenberg et al., 2008; Vellinga et al., 2013; Rautenberg et al., 2019) [36,47,48,49].

### 3.3. Turbulence Spectra Analysis and Flux Calculation

To assess the capability of the developed UAV-based EC system, the power spectral density (PSD) function of the measured wind component and scalar concentration (CO_2_ and H_2_O) are calculated and compared with the theoretical slope (−5/3) of the inertial sub-range [50]. The inertial sub-range is the region of the turbulence spectra with neither dissipation nor generation of turbulent kinetic energy, and eddies in the inertial sub-range receive energy from larger eddies and pass it on to smaller eddies [51]. If the turbulence spectra have a different shape or slope from the theoretical slope of the inertial sub-range, it may indicate a problem with the instrument, measurement setup, or data processing. In this study, we estimated the power spectral density (PSD) function using fast Fourier Transform (FFT), and then plotted the spectral intensities along the frequency domain in log–log units (where all logs use base 10). The corresponding results of turbulence spectra analysis are presented in Section 5.3.

The calculation of turbulent fluxes requires measuring the turbulence variables quickly enough to capture the details of turbulence transport on the micro-scale. In this study, the flux payloads can resolve turbulence up to a frequency of 50 Hz, allowing the length scale of turbulent fluctuations larger than 1.2 m to be detected (at a 30 m/s cruising speed). The subsequent calculation of carbon dioxide, sensible and latent heat, as well as momentum fluxes, uses the eddy covariance method and considers all the necessary corrections for open-path gas analyzers (i.e., WPL correction) [3]. The main difference between the airborne- and ground-based EC measurements is the averaging technique: the airborne EC calculates the turbulent fluctuations (wind component and associated scalar) using averages calculated over space (spatial average) rather than over time (time average) [52]. According to Crawford et al. (1993), this can introduce a bias of up to 20% in the fluxes calculated using the conventional time average for airborne EC measurement. Taking the vertical wind component w as an example, the spatial average is defined as [53]

(7) $$\bar{w}=\frac{1}{\bar{S}T}\sum_{i}w_{i}S_{i}\Delta t,$$

where S is the instantaneous ground speed of the UAV, $\bar{S}$ is the mean ground speed of the UAV, Δt is the time increment (0.02s in this study), and T is the total time.

### 3.4. Error Analysis

For the wind component measurement, the most important measurement is the vertical wind component w, which is the key to scalar flux measurements. Using the methods reported by Garman et al. (2006), the 1σ uncertainty related to the vertical wind velocity can be expressed as [54]

(8) $$\sigma_{w}=\sqrt{\sigma_{w,\alpha }^{2}+\sigma_{w,\theta }^{2}+\sigma_{w,h}^{2}},$$

(9) $$\sigma_{w,\alpha }\approx \sigma_{\alpha }U_{a},$$

(10) $$\sigma_{w,\theta }\approx -\sigma_{\theta }U_{a},$$

(11) $$\sigma_{w,w_{p}}\approx \sigma_{w_{p}},$$

where $\sigma_{\alpha }$ is the 1σ measurement precision of the attack angle (0.02° in Section 5.1), $\sigma_{\theta }$ is the 1σ precision in pitch angle of the UAV (0.1° from GPS/INS), $\sigma_{w_{p}}$ is the precision in vertical velocity (0.02 m/s, from the GPS/INS), and $U_{a}$ is the true airspeed (m/s) of the UAV.

System flux errors specific to UAV-based EC measurement were estimated using the equation provided by Mann and Lenschow (1994) [55]

(12) $$F-\langle F\left(L\right)\rangle \;\approx \;\frac{2FL_{ws}}{L},$$

where L is the length of the flight leg and $L_{ws}$ is the integral turbulent length scale inherent to each level of flight, which can be determined from the relationship of $L_{ws}\approx {\left(L_{w}L_{s}\right)}^{1/2}$, where $L_{w}$ and $L_{s}$ are the integral length of w and s, respectively, which are calculated from the autocorrelation functions.

## 4. Experimental Setup and Flight Pattern

### 4.1. Ground-Based Comparison Experiment

**Figure 3:**
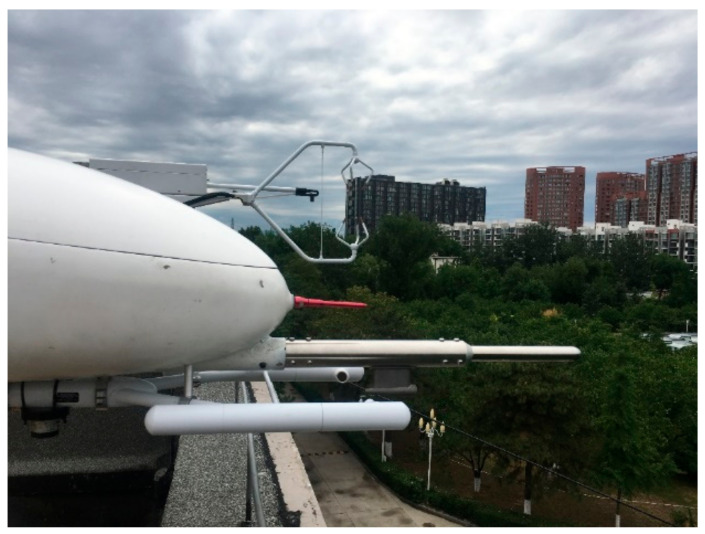
The ground-based comparison experiment between 1:00 and 6:00 p.m. on 31 May 2020 at the Chinese Research Academy of Environmental Sciences (CRAES) in Beijing ($40{}^{\circ}2^{\prime }27.51^{\prime \prime }\;N,\;116{}^{\circ}24^{\prime }45.54^{\prime \prime }\;E$) with a measurement height of 10.3 m above the ground level.

The ground-based comparison experiment was conducted between 01:00 and 06:00 p.m. on 31 May 2020, at the Chinese Research Academy of Environmental Sciences (CRAES) in Beijing, China (Figure 3). During the experiment, the predominant wind direction was from the north, with a mean wind speed of 3 m/s. The ground-based EC system is located at the roof of the Institute of Atmospheric Environment ($40{}^{\circ}2^{\prime }27.51^{\prime \prime }\;N,\;116{}^{\circ}24^{\prime }45.54^{\prime \prime }\;E$) with a measurement height of 10.3 m above the ground level. This system is composed of a CSAT3A sonic anemometer (Campbell, Logan, UT, USA) as well as an EC155 closed-path gas analyzer (Campbell, Logan, UT, USA) for sampling the wind components, sonic temperature, and CO_2_/H_2_O concentration at a frequency of 10 Hz. During the ground-based comparison experiment, we oriented the sonic anemometer and the head of the UAV to north for alignment with the orientation of the predominant wind. The turbulence probe axis of the UAV was carefully aligned parallel to the axes of the sonic anemometer with a horizontal displacement of approximately 80 and 10 cm behind of the center of the sonic anemometer. 

In the ground-based comparison experiment, we only compared the wind measurement results between the two platforms. Due to the valid angle of the 5-hole turbulence probe only being in the range of ±20° (Section 3.1), we discarded the measurements with an angle of incoming flow beyond this range, which would result in discontinuity of the measurements and would prevent flux calculations. Considering the error effect from the tilt of the sensors, the instantaneous wind vectors from the UAV and sonic anemometer were rotated using double coordinate rotation method to zero the mean vertical and cross wind components of the 30-min sample segment, which aligns the x coordinate of the reference frame of the sensor along the main streamline. Then, the instantaneous wind speed was averaged into 30-s bins, and the Pearson correlation coefficient (r) and root–mean–squared differences (RMSDs) between the UAV and sonic anemometer were calculated.

### 4.2. In-Flight Test

**Figure 4:**
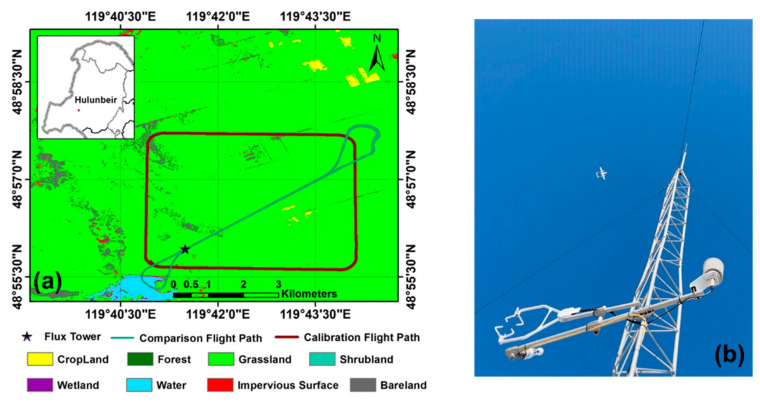
The flight path of the in-flight tests over the land cover classification map of the experimental area (**a**) and the scene photo during the comparison experiment (**b**).

Test flights were performed on 22 and 23 October 2020 at the Research Site of Field Ecological Experiments in the Hulunbeir Forest-Steppe Ecotone, which is located in the north-east of Inner Mongolia, China. The experimental area is flat, with an elevation between 630 and 700 m, and the landscape is dominated by natural grassland coverage (80%). To characterize the surface heterogeneity, the 10-m resolution global land cover data Finer Resolution Observation and Monitoring of Global Land Cover (FROM-GLC10) in 2017 was used [56]. Figure 4 displays the flight path with different flight patterns over the land cover classification map of the experimental area. 

The in-flight test included two experiments: in-flight calibration and in-flight comparison. Due to the airspace management department only allowing two to three hours for UAV flying on each day, and the ground preparation (install the aircraft in the field) of the UAV being very time-consuming, the actual flight time for flux measurement was very short. Overall, only four flights were conducted for flux measurement, while other flights were mainly used to test the safety of the integrated UAV-based EC system. The summary information on the flight pattern and meteorological conditions during the in-flight test experiment is presented in Table 3.

The in-flight calibration was used to correct the mounting misalignment between the 5-hole turbulence probe and the CG of the UAV. We used the same method as Vellinga et al. (2013) to perform the in-flight calibration that used the “box maneuver” for calibrating the offset in the heading angle ($\varepsilon_{\psi }$) and pitch angle ($\varepsilon_{\theta }$). The offset in the roll angle ($\varepsilon_{\phi }$), which was not included in the in-flight calibration, was set to 0° since its influence on the wind components is minimal. The flight path of the in-flight calibration was a box where the four orthogonal straight flight legs are flown at different heading angles for about 2 min (about 4 km) at a constant pressure altitude. During in-flight calibration, the ideal atmospheric conditions are supposed, i.e., no large turbulence transport, a constant mean horizontal wind, and a mean vertical wind near zero. The calibration values of $\varepsilon_{\psi }$ and $\varepsilon_{\theta }$ were both determined by an iteration method that finds a set of values to minimize the variance in wind speed ($\sigma_{U}$) and wind direction ($\sigma_{U_{dir}}$) to obtain a mean vertical wind component ($\bar{w}$) close to zero. We set the value of $\varepsilon_{\theta }$ to vary within the typical range of between −0.8° and −0.5°, and then iteratively calculated the mean vertical wind component ($\bar{w}$) using a step length of 0.02° to find the value of $\varepsilon_{\theta }$ for which the mean vertical wind component ($\bar{w}$) is zero. The individual straight sections of the box maneuver were used for this, and the averaged offset $\varepsilon_{\theta }$ of the four individual straight sections was used as the final result. Next, we set the possible value of $\varepsilon_{\psi }$ to vary within the range of between −3° and 1°, and iteratively calculated the horizontal wind speed using a step length of 0.5°. The final offset $\varepsilon_{\psi }$ was determined from the straight sections of the box maneuver by finding the minimum variances for horizontal wind direction ($\sigma_{U_{dir}}$) and wind speed ($\sigma_{U}$). In this paper, the calibration flight was performed under near stable atmospheric conditions above the near homogeneous grassland at 8:00–9:00 a.m. on 22 October 2020, at an average altitude of 980 m (σ=±0.9 m) above the ground level (a. g. l.). We assumed no large turbulence transport, a constant mean horizontal wind, and a mean vertical wind near zero during the in-flight calibration. 

Then, the in-flight comparison experiment was conducted by comparison with the conventional ground-based EC methods to examine the measurement performance of the developed UAV-based EC system. After we calibrated the misalignment between the turbulence probe and the CG of the UAV, several operational flights were performed horizontally in transects over the ground EC tower. The flight track was located over the central part of the selected flight region with a relative homogeneous surface, and the flight altitude was designed to be 150 m above the ground level to ensure a safe height. The flights followed some basic rules for guaranteeing the quality of measured data: the bank angle did not exceed 20°, the turn rate did not exceed 3°/s, and flight movements were performed as smoothly as possible. A ground-based EC instrument ($48{}^{\circ}55^{\prime }52.32^{\prime \prime \;}N,\;119{}^{\circ}41^{\prime }24.72^{\prime \prime \;}E$) was set up beneath the flight path to provide comparison data. The ground-based EC system is composed of a three-dimensional sonic anemometer (CSAT3, Campbell, Logan, UT, USA), and a fast-response water vapor (H_2_O) and CO_2_ density open-path infrared gas analyzer (LI7500A, LiCor, Lincoln, NE, USA), 4 m above the land surface. The sampling frequency was 10 Hz, and all the raw data were stored onto a datalogger (CR3000, Campbell, Logan, UT, USA). These data were used for a comparison of the fluxes and power spectra between the UAV- and ground-based EC measurements. The sensible heat, latent heat, and CO_2_ fluxes, as well as friction velocity from the EC tower, were computed using the conventional EC technique processes based on a 30-min averaging interval. The detailed processes followed to calculate and correct the fluxes from the EC tower can be found in the literature [57,58,59].

## 5. Results

### 5.1. Wind Tunnel Test

**Figure 5:**
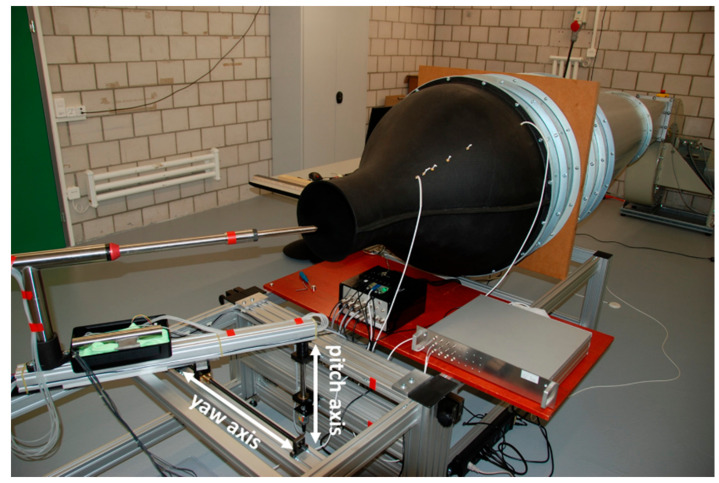
Wind tunnel calibration and testing environment for the 5-hole turbulence probe on a two-axis platform (provided by the manufacturer).

The wind tunnel calibration of the 5-hole turbulence probe was performed by the manufacturer on a two-axis platform (Figure 5) under the different measurement conditions described in Section 3.1. Then, to evaluate the residual error after the wind tunnel calibration, wind tunnel tests were repeated at various wind speed angles (α and β varied from +20° to −20° in 5° increments) and at various wind speeds (25, 30, 35, and 40 m/s), which cover the stall to dash speeds (mission speeds are typically around 30 m/s) of the UAV, with 600 s of 50 Hz data recorded for each test.

**Figure 6:**
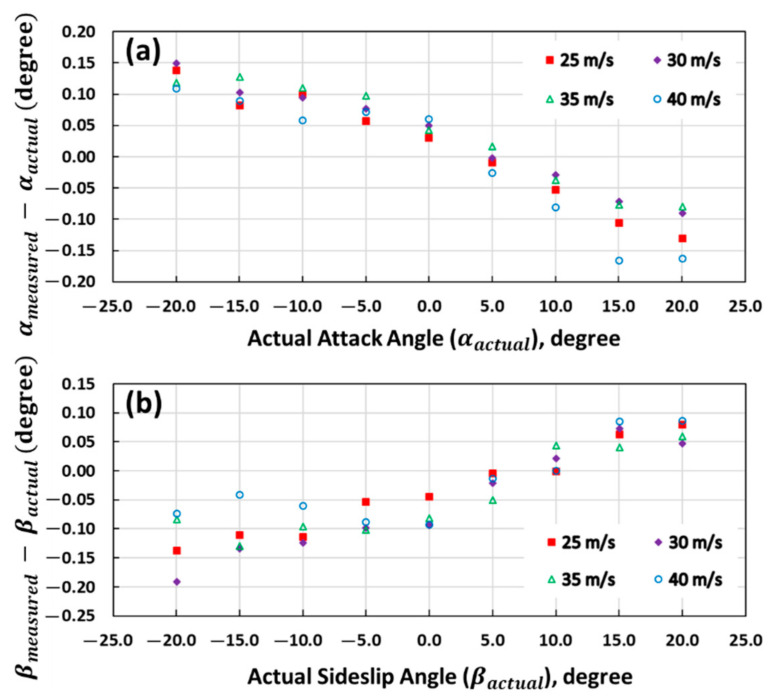
Wind tunnel test error curve for attack angle (**a**) and sideslip angle (**b**), where the *y* axis is the difference between the measured and actual angles. The inset indicates the nominal airspeeds in the wind tunnel.

The wind tunnel test results of the attack and sideslip angle are shown in Figure 6, in which the difference between the measured and actual angle is plotted as the dependent variable. As shown in Figure 6, the difference is minimal, ranging from approximately −0.17° to 0.15° for the attack angle (Figure 6a) and from approximately −0.18° to 0.09° for the sideslip angle (Figure 6b). Figure 6 also shows that the bias in $\beta_{measured}$ tended to have the same direction as $\beta_{actual}$, and presented a positive correlation with the magnitude of $\beta_{actual}$, whereas the bias in $a_{measured}$ tended to have the opposite relationship with $\alpha_{actual}$.

**Figure 7:**
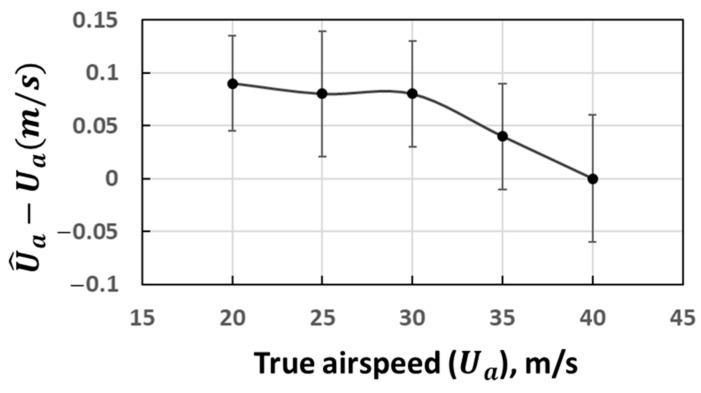
Wind tunnel test error curve for true airspeed, where the *y* axis is the difference between the measured and actual true airspeeds. For clarity, mean values with SD are shown in lieu of all datapoints.

The wind tunnel test results of the true airspeed are shown in Figure 7, in which the mean values along with their Standard Deviation (SD) of the measurement error from various attack and sideslip angles (ranging from +20° to −20°) are plotted. A small bias in ${\hat{U}}_{a}$ was observed, which varied from 0.01 to 0.08 m/s and exhibited a slight negative correlation, with the magnitude of true airspeed in the range from 20 to 40 m/s. 

These biases in the attack and sideslip angles as well as the true airspeed are not accounted for in the probe’s wind speed computation equations (Section 3.2), since these biases are typically small, as described above. We inferred that these biases were induced by the residual error after the wind tunnel calibration of the 5-hole turbulence probe, related to the calibration model used by the manufacturer. For estimating the overall precision of the vertical wind component after error propagation through the wind computation equations (Section 3.4), we estimated the overall 1σ precision of the 5-hole turbulence probe based on the wind tunnel test data, which were $\sigma_{\alpha }=\pm 0.02{}^{\circ}$, $\sigma_{\beta }=\pm 0.04{}^{\circ}$, and $\sigma_{{\hat{U}}_{a}}=\pm 0.05\;m/s$.

### 5.2. Ground-Based Comparison Test

**Figure 8:**
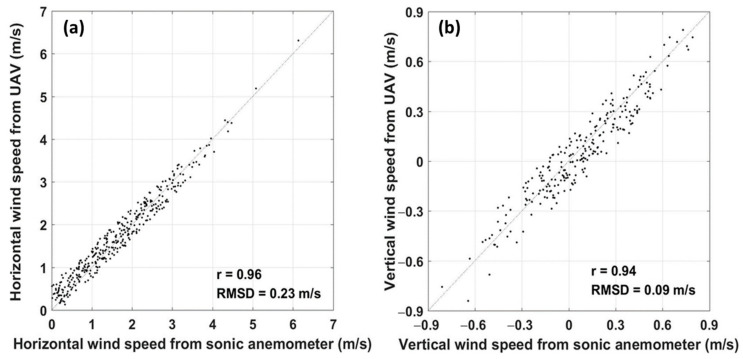
Comparison of the measured horizontal wind (**a**) and vertical wind (**b**) between the UAV and CSAT3 sonic anemometer during the ground-based comparison test on 31 May 2020. Wind vectors were calculated from the 30-s averaged instantaneous wind speed of every coordinate rotated 30-min wind sample segment. See Section 4.1 for details of the wind calculation for comparison purpose in this paper.

The comparison results of the relative horizontal wind and vertical wind from the ground-based comparison tests are presented in Figure 8, showing strong agreement between the UAV-derived and the sonic anemometer measurements (r > 0.9). The RMSDs between the wind speed measurements of the UAV and the sonic anemometer were 0.09 m/s for the vertical and 0.23 m/s for the horizontal, which are near the stated accuracies of CSAT3 (±0.08 m/s offset error ±2% reading for horizontal wind speed, and ±0.04 m/s offset error ±2% reading for vertical wind speed; https://www.campbellsci.com/csat3). The relatively large RMSDs of the wind measurements between these two methods are expected due to the range of wind speed during the ground-based comparison test being typically smaller than the scope of the calibrated airspeed of the 5-hole probe (Section 3.1). In addition, the wind tunnel test results revealed that the airspeed error of the 5-hole probe increases with the decrease in relative airspeed. We expected that the RMSDs would decrease to near the stated accuracies of the CSAT3 when the wind speed increased to near the calibrated wind speed of the 5-hole probe. Therefore, we considered that the results of the ground-based comparison experiment accurately revealed that the airframe has no influence on wind measurement and that the wind speed measured by the developed UAV-based EC system is reliable.

### 5.3. In-Flight Test

Following the above successful ground-based comparison tests, the in-flight test was performed to collect turbulence and flux data in the near-surface layer for comparison to the ground-based EC method.

#### 5.3.1. In-Flight Calibration

Due to the calibration flight being performed in the early morning and the flight altitude of 980 m above ground-level, we assumed that the calibration flight was above the local atmospheric boundary layer and under the favorable atmospheric conditions (Section 4.2).

**Figure 9:**
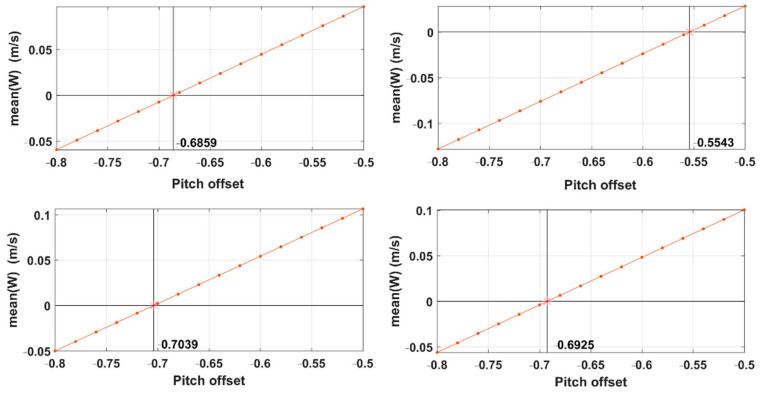
Results of determining the offset value of the pitch angle ($\varepsilon_{\theta }$) by finding the offset value that corresponded to the zero-averaged value of the vertical wind component ($\bar{w}=0$). The final offset value of the pitch angle ($\varepsilon_{\theta }$) was calculated by averaging the determined offset value from the individual straight sections of the box maneuver.

The calibration steps described in Section 4.2 were repeated several times until all calibration parameters reached a steady state. The calibration results of the offset in pitch angle ($\varepsilon_{\theta }$) using data from the straight sections of the box maneuver are shown in Figure 9. The mean vertical wind component ($\bar{w}$) of individual straight sections were calculated by varying the offset value within the typical range between −0.8° and −0.5° with a step size of 0.02°. Then, the offset value of the pitch angle ($\varepsilon_{\theta }$) corresponding to the zero mean vertical wind component ($\bar{w}=0$) was defined as the offset value of the pitch angle. Lastly, the final offset value of the pitch angle ($\varepsilon_{\theta }$) was −0.6592°, which was calculated by averaging the found offset values from the individual straight sections of the box maneuver.

**Figure 10:**
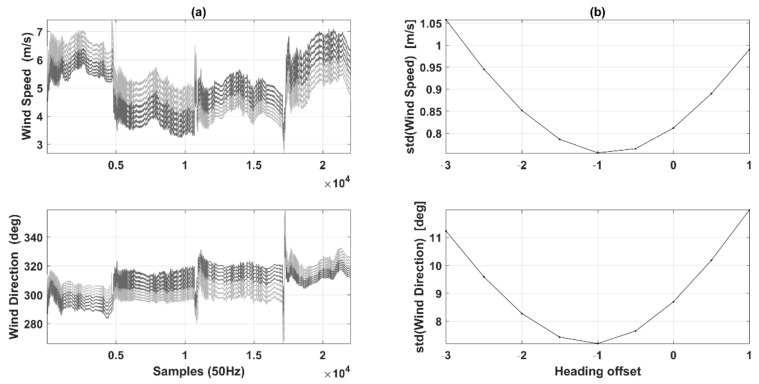
Results of determining the offset value of the heading angle ($\varepsilon_{\psi }$) by finding the offset in the heading angle ($\varepsilon_{\psi }$) with minimum variances for horizontal wind direction (**top**) and for wind speed (**bottom**) using data from box maneuver. (**a**) The influence of $\varepsilon_{\psi }$ on wind speed (**top**) and wind direction (**bottom**). (**b**) The change in variance in wind speed (**top**) and wind direction (**bottom**) within the defined possible range.

The calibration results of the offset in the heading angle ($\varepsilon_{\psi }$) are shown in Figure 10. By trying different offset values $\varepsilon_{\psi }$ under the defined possible range between −3° and 1°, we determined the offset $\varepsilon_{\psi }$ by finding the minimum variances for the horizontal wind direction ($\sigma_{U_{dir}}$) and wind speed ($\sigma_{U}$). Figure 10a shows the influence of $\varepsilon_{\psi }$ on both the calculated wind direction and the wind speed, where the dark gray lines indicate offset values of $\varepsilon_{\psi }$ smaller than its optimum, and light gray lines indicate offset values of $\varepsilon_{\psi }$ above the optimum. Figure 10b shows that $\sigma_{U_{dir}}$ and $\sigma_{U}$ reach their minima at the offset value of −1.0889° and −1.0878°, respectively. Thus, the final offset values $\varepsilon_{\psi }$ was −1.0884°, which was calculated by averaging the offset value found in $\sigma_{U_{dir}}$ and $\sigma_{U}$. Lastly, the resulting calibration constants were used for wind calculation under earth coordination.

#### 5.3.2. Spectral Analysis

**Figure 11:**
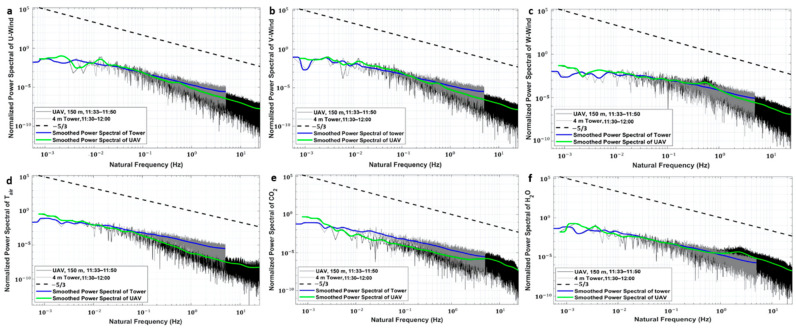
Normalized power spectra of the time series of the measured 3-D wind components in north direction (**a**), east direction (**b**), and upward direction, (**c**) as well as air temperature (**d**), carbon dioxide (**e**) and water vapor (**f**) on 23 October 2020, from the ground tower and UAV.

The normalized power spectra of the 3-D wind speed, air temperature, CO_2_ density, and H_2_O density, along with their smoothed curve for the UAV and tower measurements on 23 October 2020, are shown in Figure 11. The power spectra are presented as a function of observation frequency for evaluating the physical properties of the UAV-based EC system. The 3D wind vector for each instrument was rotated using the double rotation method for aligning the x coordinate of the reference frame of each sensor along the mean streamline. As shown in Figure 11, for all measured variables, the overall trend in the slope of the power spectral density agreed with the −5/3 law of the inertial subrange of atmospheric turbulence up to a frequency of 25 Hz. This indicated that the developed UAV-based EC system can resolve atmospheric turbulence in this part of the frequency spectrum. Some slight noise influence was identified in the variables, including horizontal and vertical wind speed (Figure 11a–c) as well as CO_2_ and H_2_O density (Figure 11e,f). The vertical wind speed and, to a lesser degree, the horizontal wind component showed a spectral peak between 0.4 and 0.8 Hz. Due to the engine and propeller of the UAV running at around 5500 rpm (revolutions per minute) during the flight, this low-frequency noise was not likely caused by the resonance from the high-frequency vibration of the UAV (engine and propeller rotating at around 90 Hz). Thus, we speculate that this spectral peak is associated with the wing’s natural frequency [60]. 

The power spectra of CO_2_ and H_2_O density exhibited a slight noise effect between 2.3 and 5.7 Hz in the high-frequency domain. This indicates that residual vibration effects remained after we added a vibration isolator on the connector between the body of the UAV and EC150. We think that the high-frequency vibration noises between 2.3 and 5.7 Hz are likely associated with the natural frequency of the rubber vibration damping we used. 

#### 5.3.3. Flight Comparison Test

**Figure 12:**
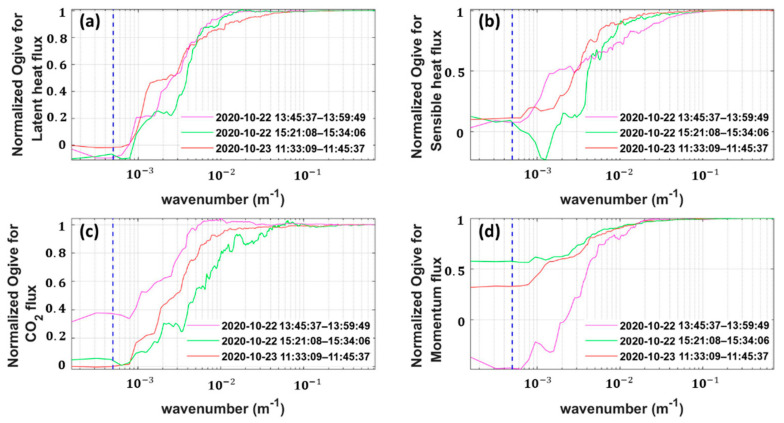
Normalized Ogive curves as a function of wavenumber for latent heat flux (**a**), sensible heat flux (**b**), CO_2_ flux (**c**) and momentum flux (**d**) from the comparison flights. The vertical black dotted line indicates the wavenumber position of the selected spatial average length of 2 km for flux calculation.

To ensure that the transect flights for comparison could capture all scales of local turbulent transport and were not contaminated by mesoscale eddies, we used the Ogive method, which is defined as the cumulative sum of a co-spectrum from low to high frequency to determine a suitable spatial average length for flux calculation [61]. The normalized Ogive curves as a function of wavenumber (1/m) for fluxes in latent heat, sensible heat, CO_2_, and momentum are shown in Figure 12. The Ogive curves display low and high wavenumber asymptotes bounding a bandwidth denoting the region in which the majority of the transport turbulent energy is captured. Figure 12 indicates that the main length scales of flux-carrying turbulence are in the range between 10 and 1600 m. According to this, we selected 2 km as the spatial average length for flux calculation. Therefore, the horizontal transect flight lines were trimmed to a length of 2 km for flux calculation in the following steps. 

To obtain the flux calculation results with statistical significance, every in-flight comparison test consisted of four legs at an altitude of 150 m above the ground level, i.e., flying back and forth twice along the predefined flight line in Figure 4. Due to the meteorological (gust wind speed larger than 12 m/s or snowfall) and airspace condition limitations, only two flight days were available and only three in-flight comparison test data were obtained. Based on the UAV data, we calculated the Monin–Obukhov (M-O) stability parameter as the indicator of atmospheric stability during the in-flight test [62]. The M-O stability parameter was 0.3 and −0.72 during the flights on 22 and 23 October 2020, respectively. This indicated that the atmospheric stability conditions were near neutral (M-O stability parameter ≈0) and unstable (M-O stability parameter < −0.2) during the two flight days, respectively [61]. A summary of the flux calculation results is presented in Table 4. The diurnal trend in the sensible heat flux, latent heat flux, CO_2_ flux, and friction wind were clearly reflected by the UAV measurements on 22 October 2020. Because of the decrease in the incident short-wave radiation in the afternoon, the values of measured fluxes at 3 p. m. are typically lower than the values measured at 1 p. m. The value of latent heat flux was typically larger than the sensible heat flux on 22 October 2020. This indicated that evaporation was significant during this period, which is reasonable because of the snowfall event the previous night. Therefore, from the trend in the UAV measurement flux data, the developed UAV-based flux measurement system can effectively capture the trend in flux change over time. 

**Figure 13:**
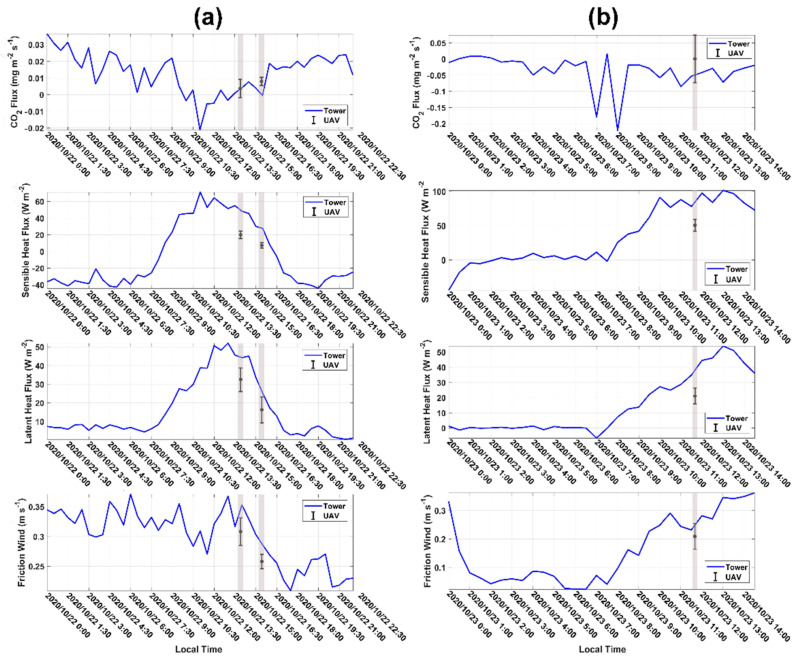
Comparison of the time series of the measured fluxes between the ground tower and UAV on (**a**) 22 and (**b**) 23 October 2020. The ground tower provides conventional 30 min averaged flux results, and the median time of each comparison flight was used to indicate the time of the measured fluxes from the UAV. Grey boxes show approximate times at which the comparison flights occurred.

The time series of conventional 30 min averaged flux data from the ground tower was used for direct comparison with the flux calculation results from the UAV (Figure 13). The median time of each comparison flight (Table 4) was used to indicate the time of the measured flux results for convenience. As shown in Figure 13, in total, the flux values measured by the UAV were typically lower than those measured by the ground tower. The sensible heat flux, latent heat flux and friction wind speed measured by the UAV were significantly underestimated compared to the ground flux tower. Assuming the flux change between half hours is linear, we obtained the ground flux observation values corresponding to the time of the UAV measured fluxes using linear interpolation. Compared with the mean value of the flux results from the UAV (Table 4), the CO_2_ flux was underestimated by about 0.02 mg/m^2^/s on average, the sensible heat flux was underestimated by about 28.1 W/m^2^ on average, the latent heat flux was underestimated by about 13.1 W/m^2^ on average, and the friction wind speed was underestimated by about 0.04 m/s on average. Generally, it is difficult to accurately assess the quality of the measured fluxes from the UAV due to the differences in sensor configuration, measurement mode, and measurement height between the ground and the airborne flux platform. As discussed in Section 6, the meteorological conditions, flux vertical divergence, inappropriate averaging length, and other factors all led to the underestimation of the fluxes measured by the UAV. Therefore, the underestimation of the measured fluxes by the UAV is considered within a reasonable range.

#### 5.3.4. Error Analysis

Firstly, we estimated the absolute uncertainty in the vertical wind component using the method of Garman et al. (2006) [54]. Based on the simplifying assumptions of straight-and-level flight, the absolute uncertainty of the vertical wind can be approximated by summing the propagated uncertainty of the individual sources (Equation (9) in Section 3.4). When the UAV flight at a true airspeed of 30 m/s (cruising speed), the calculated overall 1σ precision of the vertical wind is about 0.057 m/s, which is well within the requirements for flux measurements and is a similar performance to that reported for the BAT probe on the Sky Arrow 650 ERA [54]. The excellent precision achieved by the equipped 5-hole turbulence probe is related to its high-precision pressure sensor and the specified calibration airspeed range (the maximum is 40 m/s).

Then, we estimated the system error following the method of Mann and Lenschow (1994) [55]. We estimated the integral turbulent length scale ($L_{ws}$) using the in-flight comparison data, and the results revealed that the value of $L_{ws}$ is between 84 and 294 m. The length of each comparison flight was about 3800 m. The systematic flux error was then calculated to be between 3% and 7% for the measurements from the comparison flights. 

## 6. Discussion

In this study, we developed a UAV-based EC system for the measurement of sensible heat flux, latent heat flux, CO_2_ flux and momentum flux, as well as net radiation and photosynthetically active radiation on the regional scale. We used a high payload vertical takeoff and landing fixed-wing UAV as the platform for integration with the EC instruments. Sophisticated isolating vibration measures were applied to the contact point between the engine and the fuselage and between the EC150 and the bottom surface of the fuselage to reduce the influence of noise from the high-frequency vibrations of the engine and propeller. After completing the integration of the UAV-based EC system, several tests were performed to evaluate the performance of the developed system. This paper presented the preliminary results of the developed UAV-based EC system in wind tunnel, ground-based comparison, and in-flight comparison tests. 

The measurement of wind speed is the key to implement the eddy covariance method. The airborne EC method used a multi-hole turbulent probe to measure the wind speed based on the pressure distribution over its tip, which is the main difference compared to the ground-based EC method. Therefore, the wind measurement accuracy of the multi-hole turbulent probe is essential for the successful application of the UAV-based EC method. In this study, a 5-hole turbulence probe was equipped at the head of the UAV and was carefully calibrated by the manufacturer according to the operating environment of the UAV (the calibration range of airspeed is between 20 and 40 m/s, and the calibration range of attack and sideslip angle is ±20°). To evaluate the residual error after the calibration of the 5-hole turbulence probe, an additional wind tunnel test was conducted by the manufacturer under various wind-speed angles and wind speeds (Figure 6 and Figure 7). The results revealed some negligible biases remained in the measured attack and sideslip angles as well as the true airspeed. These biases were related to the calibration model used by the manufacturer. Based on the results of the wind tunnel test, the overall 1σ precision of the 5-hole turbulence probe was calculated (Section 5.3.1) to estimate the overall uncertainty in the vertical wind speed measurement after considering the propagation of the errors from other sensors. 

The manufacturer’s wind tunnel test was performed with the 5-hole probe alone, which cannot truly reflect the measurement performance of the 5-hole probe after mounting the probe on the UAV fuselage. The airframe would causes streamline deflection and flow deceleration in a certain range around it [35]. We placed the probe measurement tube 443.5 mm in front of the nose of the aircraft to avoid the interference effects between the airframe and the 5-hole probe. To test the synthesized performance of the wind measurement from the integrated UAV-based EC system, a ground-based comparison test was conducted using a sonic anemometer (Figure 3). Ground-based comparison experiments are not the best for testing the wind measurement performance of the UAV, mainly due to the natural wind speed being less than the calibration range of the 5-hole probe (set to between 20 and 40 m/s considering the cruise speed of UAV as being around 30 m/s). However, the comparison results revealed a strong agreement between the UAV and sonic anemometer for the measurement of horizontal wind and vertical wind speed (Figure 8). It should be noted that the 5-hold probe would achieve a higher accuracy than the current ground-based comparison test when the relative wind speed increased to the range of calibration (e.g., when the UAV is flying for observation, the typical true airspeed is above 20 m/s). Thus, the results of the ground-based comparison test demonstrated the good reliability of the wind-speed measurements from the UAV.

After ensuring that the wind-speed measurement accuracy of the UAV met the EC method requirements, a two-day in-flight test was performed to collect turbulence and flux data in the near surface layer, which were compared to the ground-based EC method. Firstly, a box-maneuvered calibration flight was conducted to correct the mounting misalignment between the 5-hole turbulence probe and the CG of the UAV in the heading ($\varepsilon_{\psi }$) and pitch ($\varepsilon_{\theta }$) angles. The ideal atmospheric conditions for calibration flight are as follows: no large turbulent transport, a constant mean horizontal wind, and a mean vertical wind near zero. Therefore, the altitude of the calibration flight was generally set to around 980 m, which is above the local atmospheric boundary layer and above the residual boundary layer, thereby avoiding the influence of the surface heterogeneity. In this study, we assumed that the calibration flight met the above requirements. However, the results from the box maneuver revealed several degrees of non-stationarity or heterogeneity in the wind field (Figure 9 and Figure 10), which may have slightly affected the determination of $\varepsilon_{\psi }$ and $\varepsilon_{\theta }$. 

After the mounting offset of the 5-hole probe was calibrated, three in-flight comparison tests were performed on 22 and 23 October 2020 by comparing with the flux results from the conventional ground-based EC method. Every comparison flight consisted of four flight legs at an altitude of 150 m above the ground level for acquiring flux results with statistical significance. The spectral properties were first investigated to evaluate the spectral response of the developed UAV-based EC system. No obvious noise influence appeared in the spectra domain of the measured variables due to the trend in the power spectrum following the −5/3 law (Figure 11). The spectral peak between 0.4 and 0.8 Hz in the horizontal and vertical wind speed is suspected to coincide with the wing’s natural frequency [60]. For the measurement of CO_2_ and H_2_O density, slight noise influences appeared between 2.3 and 5.7 Hz at high frequency (Figure 11e,f). We think that these noises are associated with the natural frequency of the rubber vibration damping we used and may be reduced or may disappear when using lower-natural-frequency rubber vibration damping in the future. Based on the results of spectral analysis, we found that (1) the measurements of wind speed and scalar concentration were not significantly influenced by the shape configuration of the UAV-based EC system, (2) the current isolating vibration measures could suitably eliminate the noise influence from the engine and propeller, and (3) the developed UAV-based flux measurement system is capable of resolving atmospheric turbulence up to a frequency of about 25Hz.

For the fluxes measurement from the UAV, the mean and the standard deviation of the results from every observation flight were calculated. The calculated flux result from the two comparison flights on 22 October 2020, revealed the time variation trend in sensible heat flux, latent heat flux, CO_2_ flux, and friction wind (Table 4). In addition, the value of latent heat flux was larger than the value of sensible heat flux, which coincided with the meteorological conditions of snowmelt. The atmospheric stability conditions of the two-flight days were near neutral (M-O stability parameter ≈0) and unstable (M-O stability parameter < −0.2) conditions, separately. The intensity of the heat fluxes (including sensible and latent flux) under near neutral conditions is weaker than under unstable conditions; accordingly, the heat fluxes (sum of sensible and latent heat fluxes) measured on 22 October 2020, is smaller than that measured on 23 October 2020. 

Theoretically, for direct comparison with surface tower fluxes, flying at low altitude during long flight legs over a uniformly homogeneous surface is desirable. The low altitude reduces vertical divergence, long legs enable capture of the low flux-contributing eddy frequencies, and the surface homogeneity simplifies horizontal flux interpretation [63]. From the comparison results between the UAV and the ground tower, the flux value was typically underestimated by the UAV, especially for the measurement of sensible heat, latent heat flux and friction wind speed (Figure 13). In this study, we assumed that the influence of the surface heterogeneity on the flux results in the comparison test was negligible due to the underlying surface of the flight region being very uniform and flat (Section 4.2). In this case, the observation altitude, and the length of the spatial average for flux calculation were the main two factors that influenced the results of the comparison. In this study, the local meaningful turbulent fluxes from UAV were estimated by defining a suitable spatial average length (2 km), using the Ogive method to exclude the influence of mesoscale turbulence (Figure 12). Mesoscale turbulence is often considered to be related to the surface heterogeneity, the influence of which needs to be minimized for comparison purposes. Due to the measurement altitude of the UAV (150 m above the ground level) being higher than the ground tower (4 m above ground level), the measurement altitude and the associated vertical flux divergence could be considered the main factors that induced the underestimation of the fluxes measured by the UAV. The influence of flux divergence on aircraft flux measurements was investigated by Vellinga et al. (2010); based on the results of flux-divergence experiments on 13 September 2007, using two small environmental research aircrafts, they found that the aircraft underestimated the sensible heat flux by about 60 W/m^2^ and underestimated the CO_2_ fluxes by about 0.35 mg/m^2^/s at a height of about 85 m above the ground when compared to the ground measurement [3]. Two other potential sources of the observed differences between the UAV and ground tower are (1) different sampling strategies and (2) inconsistency of the source areas. Ground measurement cannot adequately capture all flux contributions on as a large spatial scale as aircraft, due to its inability to conduct spatial sampling [60]. Simultaneously, the source areas of the measured fluxes from UAV are considerably larger than that from the ground tower, which may lead to the measured fluxes from the UAV being inevitably affected by surface heterogeneity [32]. Additionally, the sensor error and the flux estimation error of the ground EC may have also led to the observed differences. Especially, the traditional 30 min averaging period of the ground EC may be poorly suited for the encountered observation conditions, which requires further verification. 

One important restriction of flux measurement using UAVs is regulatory policy. The operation of airborne vehicles is regulated by law in all countries, particularly for UAV operations in the lower atmosphere over land, due to the operations of UAV posing an acute risk to other air vehicles and persons or property on the ground [64]. In China, the operation of UAV is governed by the Civil Aviation Administration of China (CAAC) [65]. UAV operation is heavily regulated in China, so a permit is required for flying a UAV when the weight of the UAV is over 150 grams, the flying altitude is higher than 120 m and flying goes beyond visual range. In this study, a permit was acquired to operate the UAV for flux measurement in the experiment region. However, during the in-flight field test, only two days were available for UAV flying due to airspace control restrictions. This dramatically limits the data acquisition ability of UAVs.

## 7. Conclusions and Further Work

In this study, we developed a UAV-based EC system for measurement of sensible heat flux, latent heat flux, CO_2_ flux and radiation fluxes, which include net radiation and photosynthetically active radiation. The atmospheric turbulence was measured fifty times per second, which means the eddies with a wavelength larger than 1.2 m can be resolved while the UAV flies at a 30 m/s cruising speed. For truly evaluating the performance of the developed UAV-based EC system, wind tunnel, ground-based comparison, and in-flight tests were carried out. This paper presented the preliminary results of those tests for investigating the measurement reliability of wind speed and turbulent fluxes from the UAV. The wind tunnel test and the ground-based comparison test with a ground sonic anemometer demonstrated that the wind speed measurement accuracy from the developed UAV-based EC system is remarkably high and meets the requirements of EC measurement. The in-flight test included calibration and comparison flights. The calibration flight was first conducted for correcting the mounting error of the 5-hole turbulence probe. Then, a 2-day field comparison test flight was performed, and the flux results were compared with the measurements from the ground tower. Despite the fluxes being underestimated by the UAV compared to the ground tower, the results revealed that the developed UAV-based EC system can effectively capture the temporal evolution in turbulent fluxes, which showed the same evolutionary trend as the ground measurements. In this study, due to the underlying surface of the flight region being very uniform and flat, the main reason for the disagreement in the flux results between the measurement platforms is the vertical flux divergence from the relatively high measurement altitude (150 m above the ground level) of the UAV. Other potential reasons for the observed differences are (1) the different sampling strategies between spatially and temporally averaging fluxes and (2) the differences in the source areas of the fluxes. Additionally, the sensor error or the estimate error in the fluxes from the ground EC may have also led to the observed differences, which requires further analysis. Lastly, based on the above results, we conclude that the developed UAV-based EC system is capable of measuring the turbulent fluxes for the conditions encountered here. 

Currently, the developed UAV-based EC system is still in the prototype stage. We aim to continue developing its data collection capability to continue learning from the detailed work undertaken by the manned aircraft EC system [15,47]. For example, the CR1000X datalogger we used will be replaced by a CR6 datalogger (Campbell, Logan, USA) to reduce the weight of scientific payloads and improve the expansibility of the system. The current vibration isolation and damping measure between the fuselage and EC150 will also be improved to eliminate the high-frequency vibration noises, which affect the CO_2_ measurement. In future improvements, a small thermal camera and a laser altimeter will be integrated in the scientific payloads to observe the surface temperature and scan the real height above the surface so that information on roughness can be obtained. More flight tests and experiments are being planned to systematically assess and improve the current UAV-based EC system. Ultimately, benefitting from the low cost and convenience of UAVs, we think that instrumented UAVs, such as that proposed in this paper, will play a more important role in collecting atmospheric measurements to help improve the understanding of the exchange mechanisms between the ecosystem and the atmosphere. 

## Figures and Tables

**Table 1:** Specification and performance of the UAV.

Mission endurance	4 h (for 10 kg payload)
Mission airspeed	25–39 m/s
Service Ceiling	3800 m
Control radio range	30–50 km
Vertical Take-off and Landing (VTOL) power	Electric motor
Level flight power	Fuel engine
Fuel	92-octane gasoline
Starting mode	Electric-starting
Fuel tank capacity	10 L
Max takeoff weight	60 kg
Payload capacity	10 kg
Payload power	User-supplied batteries
Wing span	3.7 m
Fuselage length	2.85 m
Take-off and landing mode	VTOL
Autopilot/navigation	GPS, IMU

**Table 2:** The function and sampling frequency of the scientific payload instrumentations.

Instrument	Sample Frequency	Measurement/Function	Manufacturer
Turbulence probe	50 Hz	Relative wind speed, momentum, and scalar fluxes	Simtec AG
Open path CO_2_/H_2_O Infra-red gas analyzer	50 Hz	The absolute density of H_2_O, CO_2_, and the fluxes of latent heat and CO_2_	Campbell
Platinum resistance thermometer	50 Hz	Fast temperature fluctuations, sensible heat flux	Simtec AG
thermistor	1 Hz	Temperature with high absolute accuracy, sensible heat flux	Campbell
GNSS/INS	50 Hz	Position, velocity, Georeferencing winds	Trimble
Net radiometer	10 Hz	Net radiation	Campbell
PAR radiometers	10 Hz	up-/downwelling PAR	Zipp & Zonen
Batteries	-	Power supply	-
CR1000X	-	Data acquisition, time synchronization, and power distribution	Campbell

**Table 3:** Summary of the flight pattern and the meteorological condition during the in-flight test experiment: start time (t_start), end time (t_end), horizontal length (ℓ, km), flight altitude (z, above ground level, a. g. l.), wind speed (U), and wind direction (Dir).

Flight Pattern	t_start (hh:mm:ss)	t_end (hh:mm:ss)	ℓ (km)	z (m)	U (m/s)	Dir (deg)	Description
Calibration	2020-10-22 07:31:17	2020-10-22 07:39:39	4	980	7.1	292.4	
Comparison	2020-10-22 13:45:37	2020-10-22 13:59:49	3.8	150	9.3	280.7	Consists of four horizontals transect lines.
Comparison	2020-10-22 15:21:08	2020-10-22 15:34:06	3.8	150	9.2	271.6	Consists of four horizontal transect lines.
Comparison	2020-10-23 11:33:09	2020-10-23 11:45:37	3.8	150	3.7	266.1	Consists of four horizontal transect lines.

**Table 4:** Summary of the flux calculation results of the UAV from the in-flight comparison test.

Start Time (hh:mm:ss)	End Time (hh:mm:ss)	H (W/m^2^)	LE (W/m^2^)	CO_2_ (mg/m^2^/s)	Friction Wind (m/s)
2020-10-22 13:45:37	2020-10-22 13:59:49	19.9 ± 3.2	32.4 ± 6.3	0.004 ± 0.006	0.31 ± 0.02
2020-10-22 15:21:08	2020-10-22 15:34:06	7.2 ± 4.4	16.2 ± 7.0	0.008 ± 0.002	0.26 ± 0.01
2020-10-23 11:33:09	2020-10-23 11:45:37	50.4± 8.7	21.2 ± 5.3	0.0004 ± 0.07	0.21 ± 0.05

## Data Availability

The data presented in this study are available on request from the corresponding author. The data are not publicly available due to its proprietary nature.
